# Mn_2_ Dimers Encapsulated in Silicon Cages: A Complex Challenge to MC-SCF Theory

**DOI:** 10.3390/molecules27217544

**Published:** 2022-11-03

**Authors:** Vaibhav Khanna, John Ewart McGrady

**Affiliations:** Department of Chemistry, University of Oxford, South Parks Road, Oxford OX1 3QZ, UK

**Keywords:** endohedral clusters, manganese, silicon, MC-SCF calculations

## Abstract

MC-SCF wavefunctions for three endohedral Mn/Si clusters, Mn_2_Si_10_, Mn_2_Si_12_, and [Mn_2_Si_13_]^+^, show evidence for strong static correlation, both in the Mn-Si bonds (‘in–out correlation’) and between the two Mn centers (‘up–down correlation’). We use both Restricted and Generalized Active Spaces (RAS and GAS) to place constraints on the configurations included in the trial wavefunction, showing that, particularly in the high-symmetry cases, the GAS approach captures more of the static correlation. The important correlating pairs are similar across the series, indicating that the electronic structure of the endohedral Mn_2_ unit is, to a first approximation, independent of the size of the silicon cage in which it is embedded.

## 1. Introduction

The electronic structure of endohedral silicon clusters represents a very substantial challenge to theory, primarily because the static correlation problem is complex. Strong repulsions between the metal d electrons lead to a number of closely-spaced electronic states, while the small HOMO–LUMO gaps that characterize the unsaturated Si cage also give rise to static correlation. The interactions between the metal and Si cage result in rehybridization of some of the levels, but the intrinsic challenge remains substantial [1,2]. Nevertheless, the challenge is an important one to meet because these doped clusters can be viewed as minimal models for transition metal impurities in bulk silicon, an issue of great importance in the semiconductor industry, particularly in cases where the transition metal retains some or all of its intrinsic magnetic moment. As a result, there have been many studies of a single transition metal ion inside a Si cage, dating back to the early mass spectrometry experiments of Beck and co-workers [3]. More recently, the attention of several groups, including our own, has turned to clusters containing two or more transition metals rather than one [4,5,6,7]. This is appealing from the perspective that it enhances the chances of retaining some of the intrinsic spin moment, but it also further expands the computational problem: in addition to the ’in–out’ correlation associated with the Mn-Si bonds, we now also have ’up–down’ correlation associated with the metal–metal bond. Systems with single or multiple metal–metal bonds have been explored extensively in the literature, starting with a number of seminal studies by Roos, refs. [8,9,10,11], and an adequate treatment of static correlation is often essential to their description. If we add to this the additional complexity of the M-Si and Si-Si bonds, it becomes clear that clusters of this type represent a significant challenge to theory.

X-ray crystallographic data for any M_x_Si_y_ cluster remain elusive, and techniques including photoelectron [12,13], X-ray absorption [14], and infrared spectroscopies [4,15,16] have been employed instead to provide indirect evidence about structure and bonding. All of these techniques rely, to some extent, on accurate computation to aid in their interpretation, and the tool of choice is generally Density Functional Theory (DFT), typically used to explore the potential energy surfaces of these clusters to identify low-lying isomers [4,17]. While DFT offers reasonable accuracy at low cost, there are a number of limitations associated with its use for doubly doped silicon clusters. The choice of the exchange-correlation functional is critical, and the results of these calculations often depend strongly on the choice of functional used. We confronted this problem in a recent study of Mn_2_Si_x_ clusters, where we combined infra-red multiple photon dissociation (IR-MPD) spectroscopy with DFT to explore the origins of their vibrational spectra [4]. The identification of the most stable isomer on the potential energy surface for each composition appeared to be a straightforward task, but different functionals give very different answers: LYP functionals tend to favor prism-like clusters with low vertex connectivity, while PBE and PBE0 functionals favor deltahedral structures with more highly connected vertices. There is no absolute answer to the question of which functional is correct, in this case, the isomers identified as the equilibrium structure by PBE gave a better match to the experimental spectrum than those from B3LYP, but whether this is generally true across a wider range of clusters remains an open question.

The complex correlation problem in these clusters, along with our somewhat frustrating experience of applying DFT to them, ref. [4], has encouraged us to turn to multiconfigurational SCF (MC-SCF) theory as an alternative tool to explore the electronic structure of these clusters. The problem is already complex for a single endohedral metal atom, and only a few studies have emerged on clusters of different sizes [16,18,19,20,21,22]. Amongst these, Arcisauskaite et al. investigated the geometry and electronic structure of [MnSi_12_]^+^ using DFT, IR-MPD spectroscopy, and CASPT2 [16]. For the CASPT2 calculation, Mn-Si bonding and anti-bonding orbitals were included in the (10,15) active space, along with five 4d ‘double-shell’ orbitals. The occupation numbers of active orbitals confirmed the importance of the “in–out” correlation between the electrons in Mn-Si bonds. Ngan and co-workers applied similar techniques to [MnSi_14_]^+^ [22], where a (14,15) active space captured the important static correlations. The dimensions of the active spaces here give an early insight into the problem at hand: already, for one Mn and 12 or 14 Si atoms, the active space was close to saturated. The incorporation of a second transition metal ion in, for example, Mn_2_Si_12_, will quickly push the size of the active space beyond the accessible limit; hence, some compromise will be necessary. This compromise comes in the form of restrictions on the types of excitation that are allowed within the active space. The Restricted Active Space Self-Consistent Field (RASSCF) [23] approach places restrictions on the number of excitations allowed from a set of occupied orbitals and an equivalent restriction on the excitations into a number of virtuals. In the so-called RAS2 space, all possible excitations between occupied and virtual orbitals are allowed, while only a limited number of holes are allowed in the (mostly) occupied orbitals in the RAS1 space, with the same number of excitations being allowed into the (mostly) virtual RAS3 space. The Generalized Active Space Self-Consistent Field (GASSCF) methodology, refs. [24,25,26], in contrast, divides the active space into a number of distinct subspaces, within which all possible excitations are allowed. In the limit that each GAS is made up of two electrons in two orbitals, this reduces to the so-called “separated pair” approximation [27]. In a recent study of the germanium clusters [Fe_2_Ge_16_]^4–^ and [Co_2_Ge_16_]^4–^, we applied the GAS approach to limit the active space to a manageable size and showed that both ’in–out’ (Fe/Co-Ge) and ’up–down’ (Fe/Co-Fe/Co) correlation was important [28].

**Figure 1:**
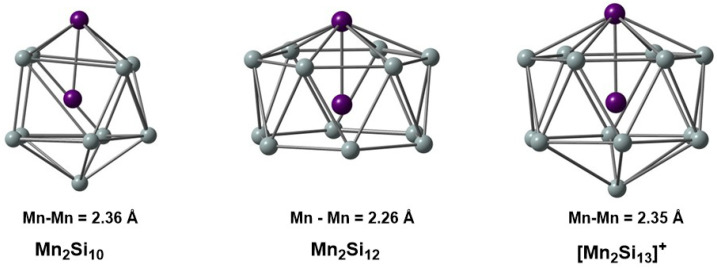
Structures of the Mn_2_Si_10_, Mn_2_Si_12_, and [Mn_2_Si_13_]^+^ clusters studied in this work. Bond lengths are given in Å.

In this contribution, we explore the electronic structure of three of the clusters that were the focus of our previous IR-MPD/DFT study [4], Mn_2_Si_10_, Mn_2_Si_12_, and [Mn_2_Si_13_]^+^ (Figure 1), using MC-SCF theory. Our goal is to gain a more robust understanding of the correlation problem in these clusters and to identify the most efficient way to partition the active space (RAS, GAS) to capture as much of the correlation energy as possible using the minimum size of the active space size. We begin with the two most symmetric cases, Mn_2_Si_12_ and [Mn_2_Si_13_]^+^, where the presence of a sixfold rotational axis aids in partitioning the correlation problem. We then return to the less symmetric Mn_2_Si_10_ to show that the same core features can be identified in the MC-SCF wavefunction, although they are slightly less transparent due to the symmetry-allowed mixing between different angular momentum quantum numbers.

## 2. Materials and Methods

**Figure 2:**
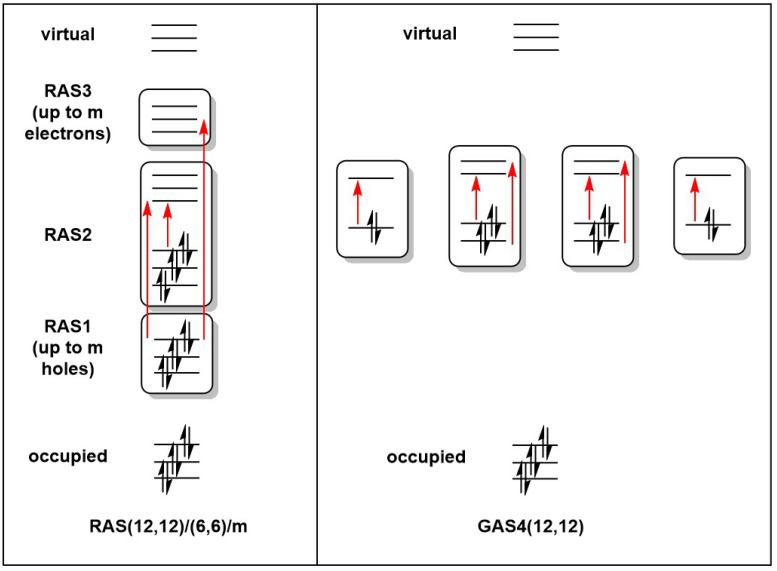
Representation of the Restricted and Generalized Active Space partitions for a (12,12) active space.

Geometries for all calculations were obtained from spin-unrestricted DFT calculations performed using the Amsterdam Density Functional (ADF) package, version 2020.103 [29]. The exchange correlation functional of Perdew, Burke, and Ernzerhof (PBE) [30] was used throughout, in conjunction with a Slater-type basis set of triple-zeta and two polarization functions (TZ2P) used on all atoms [31]. All calculations were performed using spin-unrestricted DFT. Full details of these DFT calculations were reported in our previous paper [4]. RASSCF and GASSCF calculations were performed using the OpenMolcas code [32,33], pymolcas version py2.02, with an all-electron atomic natural orbital basis set (ANO-S-VDZ) [34]. In the RAS calculations, all possible excitations were allowed within the RAS2 space, while two holes were allowed in RAS1 and two electrons in RAS3. The RAS active spaces in this work are described using the RAS(ne,no)/(ne2,no2)/m nomenclature, where ne and no are the number of electrons and orbitals in the global active space, ne2 and no2 are the number of electrons and orbitals in the RAS2 space, and m is the number of excitations allowed from/to the RAS1/RAS3 spaces (Figure 2) [35]. For the GAS calculations, all excitations were allowed within a given GAS, but no excitations were allowed between different generalized active spaces. GAS calculations are designated GASM(ne,no), where M is the number of generalized active spaces used. All RAS/GAS calculations were carried out in the largest available Abelian subgroup, which corresponded to $C_{2v}$ in the case of Mn_2_Si_12_ and [Mn_2_Si_13_]^+^ and $C_{s}$ in the case of Mn_2_Si_10_. In the first two cases, this represented a reduction in symmetry from the full $C_{6v}$ point symmetry, but the resultant wavefunction respects the full symmetry in the sense that orbitals that would be strictly degenerate in $C_{6v}$ remain degenerate despite corresponding to different irreducible representations in $C_{2v}$.

## 3. Results

### 3.1. Mn_2_Si_12_

**Figure 3:**
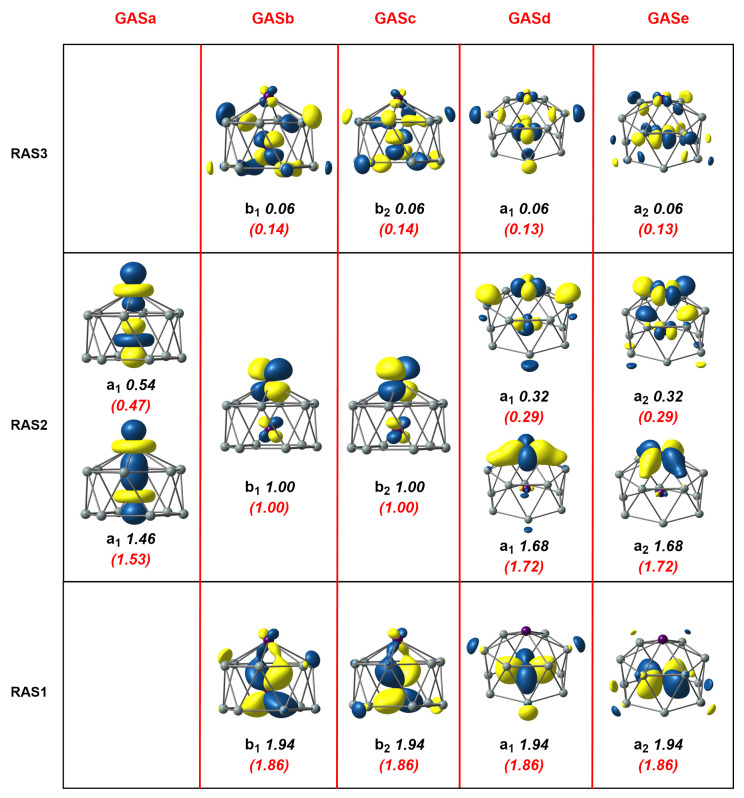
Natural orbitals and occupation numbers for the restricted active space (horizontal) and generalized active space (vertical) of Mn_2_Si_12_. Occupation numbers from the generalized active space calculation are mentioned in brackets. Calculations on Mn_2_Si_12_ used the $C_{2v}$ subgroup rather than the full $C_{6v}$ point symmetry.

An extensive series of DFT calculations reported in our previous paper [4] were unambiguous in identifying a triplet ground state, ${}^{3}A_{2}$, for this $C_{6v}$-symmetric cluster, which, as shown in Figure 1, was a hexagonal antiprismatic structure with one endohedral Mn and another capping a hexagonal face. In these unrestricted Kohn–Sham (UKS) calculations, however, the converged solution, with ${}^{3}A_{2}$ symmetry, was highly spin contaminated ($\langle S^{2}\rangle$ = 2.53, Table 1), a first clear indication of the extensive multiconfigurational character. Moreover, the degree of spin contamination was amplified if the hybrid PBE0 was used ($\langle S^{2}\rangle$ = 5.05) and the Mn-Mn bond was substantially elongated, to 2.41 Å vs. 2.26 Å for PBE (see Supplementary Materials, Table S1). We used the frontier orbitals as a reference to build an RAS(16,16)/(8,8)/2 wavefunction by placing the Mn-Mn σ bonding and anti-bonding orbitals, two Mn-Mn $\pi^{*}$ orbitals and Mn-Si bonding and antibonding δ orbitals localized primarily on the outer Mn into RAS2 (eight orbitals in total). Mn-Si π ($b_{1}$ and $b_{2}$ symmetry) and δ ($a_{1}$ and $a_{2}$ symmetry) bonding orbitals were placed into RAS1 and their antibonding counterparts into RAS3. The restricted active space was then generated by allowing up to two excitations from RAS1 and up to two electrons into RAS3, a calculation that generated 515828 configuration state functions (CSFs) in total (see Table 2). Natural orbitals and occupation numbers for the resulting ${}^{3}A_{2}$ ground-state wavefunction are collected in Figure 3, where the three RAS partitions are shown in the lower, middle, and upper horizontal boxes. The Mn-Mn $\pi^{*}$ orbitals in RAS2 contain the two unpaired electrons (occupation numbers = 1.00), consistent with the results of the unrestricted Kohn–Sham calculations reported previously [4]. The occupation numbers of 1.46 and 0.54 for the two σ orbitals in RAS2 were an immediate indication of the strength of the “up–down” correlation and were typical of a moderately strong σ bond: the effective σ bond order (EBO) of 0.46 ((1.46–0.54)/2) was substantially reduced below the formal value of 1.0. Strong “in–out” correlation was also apparent in RAS2 in the occupation numbers of 1.68 and 0.32 for the remaining Mn-Si δ orbitals that were localized primarily on the exohedral Mn center. Moving to the orbitals in RAS1 and RAS3, which were localized primarily on the endohedral Mn, the “in–out” correlation was again apparent but was now weaker, as judged by the occupation numbers of 1.94/0.06 in RAS1/3.

The RAS(16,16)/(8,8)/2 calculations suggest that the in–out correlation between Mn and Si was stronger for the exohedral Mn compared to its endohedral counterpart. This is, a priori, a reasonable finding as the exohedral Mn is coordinatively unsaturated, but it may, alternatively, simply reflect the fact that the excitations available to the electrons in the RAS1/3 were restricted. In particular, the restriction to two holes in RAS1 and two electrons in RAS3 meant that the wavefunction lacked quadruple excitations, for example, the simultaneous excitation of $\pi_{x}^{2}$$\pi_{x}^{*0}$→$\pi_{x}^{0}$$\pi_{x}^{*2}$ and $\pi_{y}^{2}$$\pi_{y}^{*0}$→$\pi_{y}^{0}$$\pi_{y}^{*2}$. These quadruples are potentially important as they are correlating electrons on the same Mn atom; indeed, they made a significant contribution to the δ and $\delta^{*}$ correlation in RAS2. In order to include these quadruple excitations, we turned to the Generalized Active Space (GAS) method, where we partitioned the same (16,16) active space into five distinct generalized active spaces labelled GASa-e (termed a GAS5(16,16) active space). These five GAS partitions, GASa-e, constitute the five columns in Figure 3, separated by red vertical lines, which contain orbitals of local σ (GASa), π (GASb,c), and δ (GASd,e) symmetry, respectively. No excitations between different partitions were allowed in the CI expansion, and the total number of CSFs that resulted was 1,142,494, approximately double the number for the RAS calculation but still within manageable limits. The natural orbitals and occupation numbers for the GAS calculation are shown in parenthesis below the RAS numbers in Figure 3. We see only marginal changes within the set of orbitals that were contained in RAS2; the occupation numbers of bonding and antibonding orbitals became larger and smaller, respectively, by 0.04 electrons in the case of the δ orbitals and 0.07 electrons for σ. The numbers for the orbitals that were in RAS1 and RAS3, in contrast, decreased/increased by 0.08 electrons, indicating that the simultaneous excitations of the four π or the four δ electrons made a substantial contribution to the overall correlation. This improved description of the correlation was reflected in the calculated correlation energy, which was 0.038 au (1 eV) lower for the GAS calculation. Finally, we note that we reduced the number of GAS partitions to three (GAS3(16,16)) by merging GASb and GASc to form a π’ GAS and GASd and GASe to form a ’δ’ GAS. This came at the expense of a more than threefold increase in the number of CSFs (now 3,708,680) but with only a marginal further gain in correlation energy (0.1548 $E_{h}$ vs. 0.1535 $E_{h}$ for GAS5(16,16). The cross excitations $\pi_{x}^{2}$$\pi_{y}^{*0}$→$\pi_{x}^{0}$$\pi_{y}^{*2}$ clearly had negligible amplitude in the MC-SCF wavefunction due to symmetry, and the ability to eliminate these from the trial GAS wavefunction is a powerful feature of the GAS methodology in systems with axial symmetry.

### 3.2. [Mn_2_Si_13_]^+^

**Figure 4:**
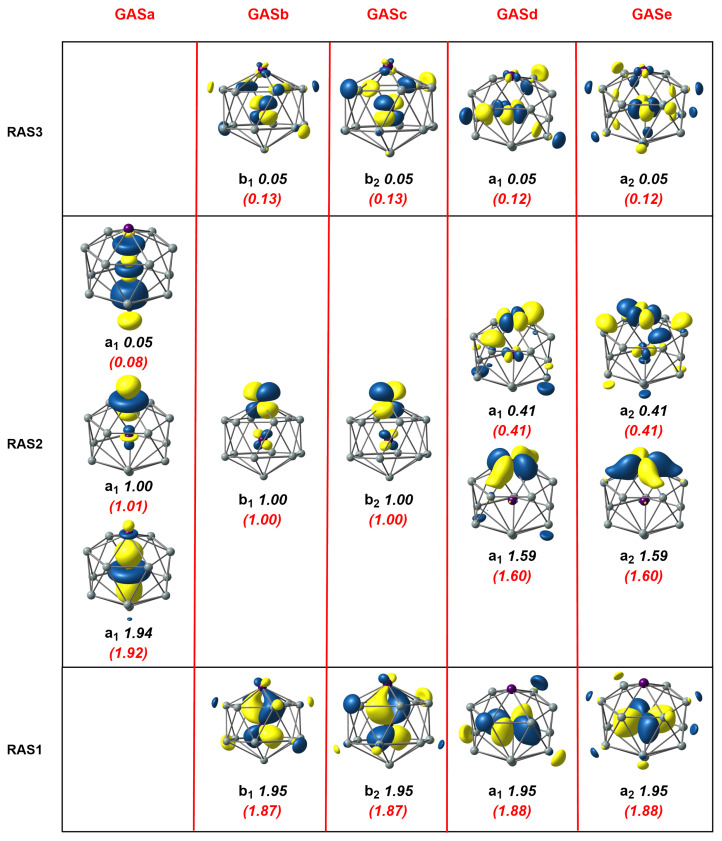
Natural orbitals and occupation numbers for the restricted active space (horizontal) and generalized active space (vertical) of [Mn_2_Si_13_]^+^. Occupation numbers from the generalized active space calculation are mentioned in brackets. Calculations on [Mn_2_Si_13_]^+^ used the $C_{2v}$ subgroup rather than the full $C_{6v}$ point symmetry.

Moving to the second cluster in our study, the [Mn_2_Si_13_]^+^ cation, the axial symmetry shown in Figure 1 already leads us to anticipate that the GAS methodology will present advantages. Our previous DFT study [4] identified a $C_{6v}$-symmetric quartet, ${}^{4}A_{2}$, which was derived, at least conceptually, from the Mn_2_Si_12_ antiprism by capping the remaining hexagonal face with a Si^+^ ion. We approached the MC-SCF problem in the same way as for Mn_2_Si_12_, seeking first to define a RAS1/2/3 space, summarized in the three rows of Figure 4. The RAS(17,17)/(9,9)/2 wavefunction shown in Figure 4 was qualitatively similar to that for Mn_2_Si_12_, the most obvious difference being that there were three single occupied orbitals in RAS2, two of π symmetry and one σ, all localized on the exohedral Mn. The correlation within the δ/δ* orbitals in RAS2 was very similar to before, as judged by the occupation numbers. Likewise, the correlations involving the endohedral π and δ orbitals in RAS1/3 were almost identical to those in Mn_2_Si_12_. The most conspicuous difference appeared in the lower correlation in the σ set (occupations of 1.94 and 0.05 compared to 1.46/0.54 in Mn_2_Si_12_), which was a consequence of the presence of an unpaired electron in the Mn-Mn $\sigma^{*}$ orbital, which blocked 2-electron excitations from Mn-Mn σ. The correlating virtual was, instead, a σ orbital with Mn 4$d_{z^{2}}$ character (one of the so-called ’double-shell’ set), with less effective correlation as a result. The alternative GAS5(17,17) wavefunction (the GAS are collected in the columns of Figure 4) again captured more ’in–out’ correlation between the endohedral π and δ orbitals in RAS1 and RAS3 due to the introduction of quadruple excitations, and this led to a stabilization of the MC-SCF wavefunction by 0.0799 au (2.17 eV), at the cost of increasing the number of CSFs from 1,632,392 in the RAS wavefunction to 3,469,636 in the GAS.

### 3.3. Mn_2_Si_10_

**Figure 5:**
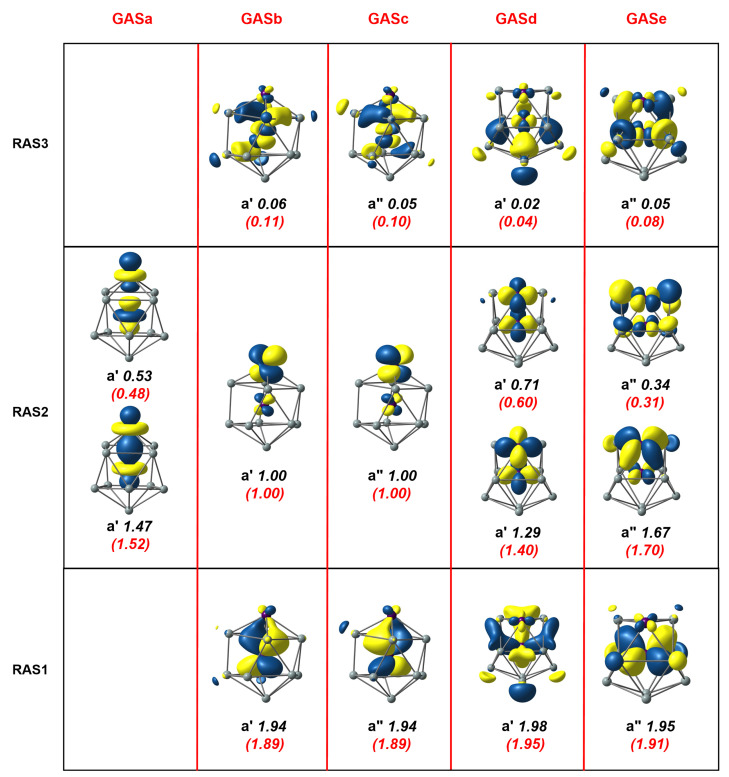
Natural orbitals and occupation numbers for the restricted active space (horizontal) and generalized active space (vertical) of Mn_2_Si_10_. Occupation numbers from the generalized active space calculation are mentioned in brackets. Calculations on Mn_2_Si_10_ used the $C_{s}$ point group.

Our previous DFT calculations indicated a $C_{s}$-symmetric triplet ground state (${}^{3}A^{\prime \prime }$) for Mn_2_Si_10_ [4], where again the UKS solution was highly spin contaminated ($\langle S^{2}\rangle$ = 2.95, see Table 1). The identification of RAS and GAS wavefunctions was in this case complicated somewhat by the lower symmetry of the system, which precluded a clean separation of σ, π, and δ based on the representations of the group alone. Nevertheless, through manipulation of the initial orbitals, we identified an RAS(16,16)/(8,8)/2 active space that bore close resemblance to that for Mn_2_Si_12_, Figure 5. The RAS2 space contained two singly-occupied Mn-Mn $\pi^{*}$ orbitals along with the Mn-Mn σ bonding/antibonding pair and the two Mn-Mn bonding/antibonding δ orbitals. Pairs of Mn-Si π and δ bonding/antibonding character were then placed in RAS1 and RAS3, respectively, with two excitations from RAS1 and up to two electrons in RAS3 allowed. The occupation numbers of 1.47 and 0.53 for the two σ orbitals in RAS2 indicated an “up–down” correlation of very similar magnitude to that in Mn_2_Si_12_. Likewise, correlation within the $\delta -\delta^{*}$ pair of $a^{\prime \prime }$ symmetry (1.67/0.34) was similar to that noted in the previous two clusters. In contrast, the δ pair of $a^{\prime }$ symmetry was fundamentally different in nature: rather than reflecting an ‘in–out’ correlation between Mn-Si bonding–antibonding pair, it reflected an ‘up–down’ Mn-Mn δ−$\delta^{*}$ correlation, with occupation numbers of 1.29 and 0.71, indicating the presence of a very weak Mn-Mn δ bond. The reasons for this distinction between the orbitals of Mn-Mn δ symmetry in Mn_2_Si_10_ compared to the other two clusters stemmed from the unique features of the coordination environment of the exohedral Mn ion, which capped a 4-membered ring in Mn_2_Si_10_ but 6-membered rings in both Mn_2_Si_12_ and [Mn_2_Si_13_]^+^. The approximate fourfold symmetry of the coordination environment meant that one of the two exohedral Mn δ orbitals must be approximately Mn-Si nonbonding, while for the sixfold symmetric coordination, both components could engage in Mn-Si bonding (and indeed were degenerate). The absence of a Mn-Si overlap for one δ component then allowed for the formation of the Mn-Mn δ bond. In the GAS partition, we again found a relative stabilization of the MC-SCF wavefunction of 0.0448 au (1.22 eV), at the cost of a marginal increase in the number of CSFs.

## 4. Conclusions

We used the MC-SCF methodology to explore the trends in static electron correlation for a series of three clusters, all of which contained a Mn_2_ dimer unit, partially encapsulated by Si cages of increasing size. We used both restricted and generalized active space approaches (RAS and GAS) to partition a (16,16) (in the case of Mn_2_Si_10_, Mn_2_Si_12_) or (17,17) ([Mn_2_Si_13_]^+^) active space, thereby placing restrictions on the type of excitations that entered the trial wavefunction. In the RAS approach, orbitals were placed in RAS1, RAS2, or RAS3 based on the strength of the correlations, while in the GAS approach the orbitals were separated by symmetry into five groups with Mn-Mn σ, $\pi_{x}$, $\pi_{y}$, $\delta_{xy}$, and $\delta_{x^{2}-y^{2}}$ character. The GAS approach, unlike RAS, allowed for 4-electron excitations from orbitals of δ and π symmetry on the endohedral Mn, a difference that was reflected in the lower SCF energies obtained by the GAS approach and greater deviations of the natural occupation numbers from 2.0 and 0. Across the series of three clusters, the nature of the static correlation was remarkably constant, suggesting that the electronic structure of the Mn_2_ unit was largely independent of the size of the Si cluster in which it was embedded. The clusters showed strong ‘in–out’ correlation of the Mn-Si bonding electrons that were qualitatively similar to those noted in [MnSi_12_]^+^, as well as substantial ‘up–down’ correlation of the electrons involved in Mn-Mn bonding. The complex static correlation picture revealed by these calculations was consistent with the very variable picture presented by DFT, where the most stable structure predicted was highly dependent on the choice of functional, and hence, the details of description of exchange and correlation [4].

## Figures and Tables

**Table 1:** Mulliken spin densities and $<S^{2}>$ values of Mn_2_Si_10_, Mn_2_Si_12_, and [Mn_2_Si_13_]^+^ computed with PBE and PBE0 functionals.

Cluster	ρ $\left({\mathrm{Mn}}_{\mathrm{exo}}\right)$		ρ $\left({\mathrm{Mn}}_{\mathrm{endo}}\right)$		ρ $\left({\mathrm{Si}}_{x}\right)$		$<S^{2}>$	
	PBE	PBE0	PBE	PBE0	PBE	PBE0	PBE	PBE0
Mn_2_Si_10_	3.00	4.56	−0.58	−2.56	−0.41	0.00	2.95	5.01
Mn_2_Si_12_	2.83	4.24	−0.22	−2.73	−0.61	0.49	2.53	5.05
[Mn_2_Si_13_]^+^	3.41	4.14	0.67	2.18	−1.08	−3.33	4.31	6.01

**Table 2:** Total energies (*E*) and number of configuration state functions (CSFs) obtained from Hartree Fock (HF), restricted (RAS), and generalized (GAS) active space calculations for Mn_2_Si_10_, Mn_2_Si_12_, and [Mn_2_Si_13_]^+^. All energies are given in Hartree units.

Cluster	HF	RAS			GAS		
	*E*	CSFs	*E*	*E* ${}_{\mathrm{corr}}$	CSFs	*E*	*E* ${}_{\mathrm{corr}}$
Mn_2_Si_10_	−5188.2275	1,031,552	−5188.4276	0.2001	1,142,494	−5188.4724	0.2449
Mn_2_Si_12_	−5766.0617	515,828	−5766.1772	0.1155	1,142,494	−5766.2152	0.1535
[Mn_2_Si_13_]^+^	−6054.6867	1,632,392	−6054.7848	0.0981	3,469,636	−6054.8647	0.1780

## Data Availability

The data presented in this study are available in Supplementary Materials.

## Supplementary Materials

The following supporting information can be downloaded at: https://www.mdpi.com/article/10.3390/molecules27217544/s1, Cartesian coordinates of the geometries used.

## Abbreviations

The following abbreviations are used in this manuscript:

DFT	Density Functional Theory
MC-SCF	Multiconfigurational Self-Consistent Field
