# Supplementary material for: Mn2 Dimers Encapsulated in Silicon Cages: A Complex Challenge to MC-SCF Theory
Source: Molecules. 2022 Nov 3;27(21):7544. doi: 10.3390/molecules27217544 (PMC9658461; doi:10.3390/molecules27217544)
Supplement: Supplementary file 1 [file molecules-27-07544-s001.zip › molecules-1977868-supplementary.pdf]

Supporting information

Mn_2 dimers encapsulated in silicon cages: a complex challenge to MC-SCF theory

Vaibhav Khanna and John E. McGrady

Department of Chemistry, University of Oxford, Oxford, OX1 3QZ, United Kingdom

Functional dependence of Mn-Mn bond lengths in Mn_2Si_{10}, Mn_2Si_{12} and $[Mn_2Si_{13}]^+$

In order to explore the functional dependence of geometry, and in particular the Mn-Mn bond length, we have re-optimised the geometries using hybrid functionals (B3LYP, PBE0), a metaGGA (SCAN) and a metahybrid (TPSSh).

Table S1 Optimized Mn-Mn bond lengths (in Angstrom) in Mn_2Si_{10}, Mn_2Si_{12} and $[Mn_2Si_{13}]^+$ using the PBE, SCAN, B3LYP, PBE0 and TPSSH functionals.

	PBE	SCAN	B3LYP	PBE0	TPSSh
Mn_2Si_{10}	2.36	2.38	2.44	2.42	2.37
Mn_2Si_{12}	2.26	2.28	2.40	2.41	2.27
$[Mn_2Si_{13}]^+$	2.35	2.36	2.44	2.42	2.36

Cartesian coordinates used in RAS/GAS calculations on Mn_2Si_{10}, Mn_2Si_{12} and $[Mn_2Si_{13}]^+$ (optimized with the PBE functional)

Mn_2Si_{10}

Mn	0.11795933	-0.11381712	-0.00000000
Mn	2.46729237	0.13738000	-0.00000000
Si	-1.07737015	0.58436286	2.03365367
Si	1.06641812	1.55021925	1.37903040
Si	-0.99480515	-1.79119004	1.26655830
Si	-1.07737015	0.58436286	-2.03365367
Si	-0.99480515	-1.79119004	-1.26655830
Si	1.40583470	-1.42508757	1.43245794
Si	-1.00444419	2.10847172	-0.00000000
Si	1.06641812	1.55021925	-1.37903040
Si	1.40583470	-1.42508757	-1.43245794
Si	-2.38096254	0.03135638	-0.00000000

Mn_2Si_{12}

Mn	0.00000000	0.00000000	-0.40922965
Mn	0.00000000	0.00000000	1.84690040
Si	2.10442167	-1.21498842	-1.37422324
Si	2.10442167	1.21498842	-1.37422324
Si	0.00000000	-2.42997684	-1.37422324
Si	0.00000000	2.42997684	-1.37422324
Si	-2.10442167	-1.21498842	-1.37422324
Si	-2.10442167	1.21498842	-1.37422324
Si	2.41883792	0.00000000	0.87072311
Si	1.20941896	2.09477509	0.87072311
Si	1.20941896	-2.09477509	0.87072311
Si	-1.20941896	2.09477509	0.87072311
Si	-1.20941896	-2.09477509	0.87072311
Si	-2.41883792	0.00000000	0.87072311

$[\mathbf{Mn_2Si_{13}}]^+$

Mn	0.000000000	0.000000000	0.380000000
Mn	0.000000000	0.000000000	-1.972152290
Si	2.048763690	-1.182854270	-0.823965660
Si	2.048763690	1.182854270	-0.823965660
Si	0.000000000	-2.365708530	-0.823965660
Si	0.000000000	2.365708530	-0.823965660
Si	-2.048763690	-1.182854270	-0.823965660
Si	-2.048763690	1.182854270	-0.823965660
Si	2.446465770	0.000000000	1.344989400
Si	1.223232880	2.118701500	1.344989400
Si	1.223232880	-2.118701500	1.344989400
Si	-1.223232880	2.118701500	1.344989400
Si	-1.223232880	-2.118701500	1.344989400
Si	-2.446465770	0.000000000	1.344989400
Si	0.000000000	0.000000000	2.651539260
